# The quantitative evaluation of the impact of viable medial meniscus graft type on the biochemical and biomechanical properties of the rabbit tibial cartilage

**DOI:** 10.1186/s13018-015-0311-8

**Published:** 2015-11-12

**Authors:** Tomasz J. Zwierzchowski, Jolanta Janus, Włodzimierz Konecki, Grzegorz Kubiak, Jarosław Fabiś

**Affiliations:** Department of Arthroscopy, Minimally Invasive Surgery and Sports Traumatology, Medical University of Lodz, ul. Żeromskiego 113, 90-549 Łódź, Poland; Department of Pathophysiology, Medical University of Lodz, ul. Narutowicza 60, Łódź, Poland; Department of Fibre Physics and Textile Metrology, Technical University of Lodz, ul. Żeromskiego 113, 90-549 Łódź, Poland

**Keywords:** Cartilage biochemical and biomechanical properties, Viable meniscal transplantation

## Abstract

**Background:**

Knowledge of the impact of viable medial meniscus allograft and autograft transplantation on biochemical and mechanical properties of cartilage is needed to understand the development of joint osteoarthritis. The purpose of this study was to evaluate this relationship 6 months after viable medial meniscal autograft and allograft transplantation.

**Methods:**

Twenty rabbits were chosen for the study. The medial menisci were excised from 14 animals and stored under tissue culture conditions for 2 weeks. Seven menisci were implanted as autografts (group A) and seven as allografts (group B). The control group consisted of six animals which underwent arthrotomy. The tibial cartilage was used for mechanical and biochemical evaluation.

**Results:**

The respective decreases of glycosaminoglycans (GAGs) and elasticity were 13.4 and 14.8 % for group A and 30.4 and 32.6 % for group B. The differences between group A and B and between each group and the control were statistically significant. The total collagen content was significantly lower in group B.

**Conclusions:**

The type of viable meniscal graft has an influence on the biochemical composition of the extracellular matrix (ECM) and biomechanical properties of the underlying tibial cartilage. A 1 % decrease of glycosaminoglycan content is associated with a 1.1 % decrease of cartilage elasticity. The average ratio of decrease of cartilage elasticity to that of the meniscus was 0.77 regardless of the type of meniscus graft. The viable allograft causes irreversible ECM disorder of the cartilage. Knowledge of the biochemical composition of the ECM meniscal grafts may serve as a predictor of their chondroprotective properties.

## Background

One of the most common causes of knee cartilage degeneration is an injury of the meniscus [1]. In the case of an irreversible loss of the meniscus, allograft transplantation or scaffold implantation is needed to prevent cartilage destruction and the early development of osteoarthritis [2, 3]. Although allografts are known to play a chondroprotective role, osteoarthritic changes are still present. Hence, Verdonk et al. have introduced the viable meniscus transplantation procedure, which represented a considerable step forward in the reduction of negative impact of meniscal transplant storage on their biological value: fresh meniscus allografts are incubated for 2 weeks under tissue culture to enhance the cartilage support [4]. Zwierzchowski et al. have reported that this procedure has no impact on the molecular, biochemical, or mechanical properties of graft in a rabbit model of viable meniscus transplantation [5]. Furthermore, they disclosed that the type of graft is responsible for biological incorporation, the reconstruction of the meniscus tissue, and ultimately, its molecular, biomechanical, and mechanical properties, thus indicating some of the unfavorable findings of Verdonk et al. [4, 6].

The modern concept of tissue engineering offers the promise of implants with biomechanical and biological properties resembling natural meniscus, which will be prepared in the near future [7]. However, so far, little is known about the quantitative impact of the biochemical and mechanical properties of the type of the meniscus grafts on biochemical and mechanical properties of the tibial articular cartilage after their transplantation [3, 8]. Our previous experimental studies indicate the presence of homeostatic disorders of the tibial hyaline cartilage, manifested by excessive apoptosis of chondrocytes 6 months after viable rabbit medial meniscus autograft and allograft transplantation and an increased expression of collagenase-1 (MMP-1), stromelysin-1 (MMP-3), and the tissue inhibitor of metalloproteinases-2 (TIMP-2) after allograft transplantation [9]. As excessive apoptosis and higher metalloproteinase expression were found to accompany the altered biochemical and biomechanical properties of the implanted menisci, it can be assumed that the biochemical and biomechanical properties of the hyaline cartilage might be also altered in a similar pattern [6].

Therefore, further studies have been performed aimed at identifying the quantitative impact of the biochemical and biomechanical properties of transplanted rabbit meniscus autografts and allografts on the biochemical and mechanical properties of the tibial cartilage which is covered by them. Thus, three study hypotheses arise: (1) the biochemical and biomechanical properties of the type of viable meniscal graft have an influence on the biochemical composition of the extracellular matrix (ECM) and the mechanical properties of the underlying tibial cartilage, (2) the biochemical composition of the ECM of the cartilage has a direct impact on its biomechanical properties, and (3) the knowledge of the biochemical composition of ECM meniscal grafts may serve as a universal predictor of their chondroprotective properties.

## Methods

### Study design

Twenty-two white male New Zealand rabbits, body weight 3500–4000 g, were chosen for the study. All procedures performed in the study involving animals were in accordance with the ethical standard of the institution or practice at which the study was conducted. Two study groups were distinguished: eight animals with autografts (group A) and eight with allografts (group B). The third was a control group consisting of six animals which underwent arthrotomy (sham operations). The excised left knee medial menisci (from 16 animals) were stored under tissue culture conditions. After 2-week storage, to replicate clinical conditions, eight animals were implanted with own autografts and eight with donor allografts. Two rabbits (one with autograft and one with allograft) developed infective arthritis and were excluded from the study. All animals were euthanized after 6 months; the tibial cartilage was used for biomechanical examination and biochemical analysis. Biomechanical and biochemical evaluation was performed by a single observer who was blinded to the experimental groups.

### Surgical procedure

All surgical procedures were performed by the same surgeon. The rabbits were premedicated with intravenous medetomidyne at a dose of 0.01 ml/kg b.w. and atropine at a dose of 0.05 mg/kg b.w. To maintain anesthesia, Bioketan (ketamine) was administered as an intramuscular dose of 3 mg/kg b.w. Antisedan at a dose of 0.01 ml/kg b.w. was used to wake the animals. Under aseptic conditions, a skin incision of about 25 mm was performed on the medial side, and two medial anterior and posterior openings were made in the left joint capsule. The medial meniscus was excised according to the modified Shibuya technique without cutting the collateral tibial ligament [9]. The wound was closed in layers. The excised meniscus was placed in a tissue culture bottle (Nunk) with Eagle medium (DNEM: Ham F12 (1:1), Gibco) with the addition of 10 % fetal calf serum (Sigma) and 1 % penicillin, streptomycin, and fungizone (Gibco). The containers were stored in an incubator for 2 weeks, at 37 °C in an atmosphere of 5 % CO2 and 95 % air. Every 3 days, the bottles and medium were exchanged. From among the menisci prepared in this way, eight rabbits were implanted with their own menisci into the left knee joint—autografts. The remaining eight animals were implanted with donor menisci—allografts. The medial meniscus was implanted according to a modified technique of Sommerlath and Gillquist [10]. After each procedure, antibiotic (enrofloxacin) was administered for prophylactic purposes at an intramuscular dose of 10 mg/kg b.w. every 12 h for 3 days. After surgery, the animals received butorphanol subcutaneously (0.3 mg/kg b.w.). No plaster immobilization was applied, and the rabbits were allowed to move freely in standard cages. The animals’ behavior was observed systematically. Postoperative wounds healed per primam, except two animals, which were excluded from the study.

After 6 months, the animals were euthanized by administering a lethal dose of pentabarbitone sodium intravenously. The knee joint was opened in the left limb. The hyaline cartilage from medial tibial compartments was carefully excised from all rabbits, placed in containers with tissue culture media, and immediately examined mechanically and biochemically. For mechanical and biochemical studies, the hyaline cartilage from meniscus-covered areas was harvested.

### Biomechanical test

The mechanical evaluation of the tibial hyaline cartilage was based on a stress-relaxation test [11, 12]. Immediately after harvesting, the cartilage samples were weighed and subjected to a compression test on a floor model INSTRON TT-BM testing instrument. The cartilage sample was placed in a specially designed metal nonporous matrix with a shape of the harvested cartilage, which prevented the specimen from shifting laterally during compression. The cartilage sample was compressed with a force of 2.5 N, held for 5 min, and allowed to relax. The degree of elasticity, calculated from elastic and total elongation, was determined from the obtained hysteresis curve. The mechanical parameters were determined based on our previous mechanical examinations of the rabbit’s meniscus [6].

### Biochemical evaluation

Directly after mechanical testing, the cartilage samples were dried, weighed, and subjected to biochemical analysis. The water content was calculated by subtracting the dry weight from the wet weight of the cartilage. The colorimetric method described by Woessner was used to assess the hydroxyproline fraction [13]. The total collagen content was derived from hydroxyproline by multiplying value by a factor of 7.6 suggested by Venn and Maroudas [14]. The determination of total sulfated glycosaminoglycans (GAGs) content was based on the method described by Ferndale et al. [15].

### Statistical methods

All values were expressed as the mean ± SD (standard deviation). All the variables were treated as measurable ones. Shapiro-Wilk’s normality test served as a basis for deciding on a parametric or nonparametric statistical test. In all groups, there was no reason to dismiss the hypothesis that the variables have a normal distribution. Shapiro-Wilk’s normality test results enabled the application of parametric methods in the subsequent calculations. Next, Levene’s test was conducted, which dismissed the hypothesis of the variance homogeneity for the biochemistry and the biomechanics. An ANOVA variance analysis test was used for the evaluation of statistical differences between experimental and control groups.

*p* < 0.05 was considered to be the level of statistical significance.

## Results

### Biomechanical test

**Fig. 1:**
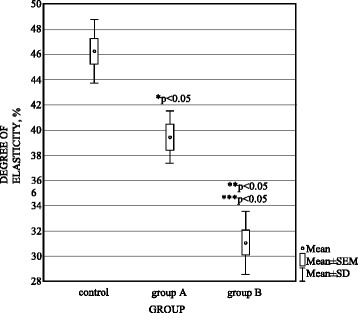
Degree of elasticity (%) in control, group A (autografts), and group B (allografts). *p < 0.05 between group A and control. **p < 0.05 between group B and group A. ***p < 0.05 between group B and control

The cartilage degree of elasticity was seen to be lower in group A (meniscal autografts) (*p* < 0.05) (39.37 ± 2.04) in relation to the controls (46.22 ± 2.51) by a statistically significant difference. The highest decrease was observed in group B (meniscal allografts) (31.15 ± 2.50) with statistically significant difference between group B and controls and group A (Fig. 1)

### Biochemical analysis

The greatest changes in the biochemical composition of the cartilage extracellular matrix occurred in group B and concerned all parameters studied. The values of biochemical parameters in the study groups and in the controls are given in Table 1.

**Table 1:** Water, glycosaminoglycans (GAGs), hydroxyproline, total collagen content, and glycosaminoglycans/hydroxyproline ratio in control and study groups

	Water (%)	GAGs (Glycosaminoglycans) (μg/mg dry mass)	Hydroxyproline (μg/mg dry mass)	Total collagen (μg/mg dry mass)	Glycosaminoglycans/hydroxyproline ratio
Control group	77.40 ± 2.51	139.19 ± 3.39	102.83 ± 1.01	781.49 ± 7.70	1.35 ± 0.30
Group A/autografts	82.65 ± 2.72^a^	120.58 ± 4.13^a^	102.07 ± 1.07	775.71 ± 7.94	1.18 ± 0.25^a^
Group B/allografts	89.62 ± 2.62^bc^	96.84±6.35^bc^	88.11±0.86^bc^	669.64±6.51^bc^	1.10±0.14^bc^

Mean value ± standard deviation (SD)

^a^ Statistically significant difference between control and group A (*p* < 0.05)

^b^ Statistically significant difference between control and group B (*p* < 0.05)

^c^ Statistically significant difference between group A and group B (*p* < 0.05)

## Discussion

This is the first study to document the biochemical composition of the ECM and the biomechanical properties of the cartilage after the transplantation of viable meniscus autografts and allografts, depending on the type of graft. These results confirm the observations of Lopez et al. and Pennock et al. regarding the close relationship between changes in the articular cartilage and the menisci at the onset of secondary osteoarthritis [16, 17]. A decrease of collagen and GAGs contents and an increase of water content are characteristic features of the degenerative process in the hyaline cartilage leading to the decrease of biomechanical properties and should be regarded as highly unfavorable for joint functioning [14, 18–22].

The uniqueness of our study depends on the possibility to correlate the changes in the biochemical and biomechanical properties of the cartilage with those recorded in the menisci as well as the presence of apoptosis and MMP1,3 and TIMP2 content within these structures (Table 2) [6]. All these studies together show that the decrease of collagen content within the meniscus and cartilage is connected with the allograft due to the presence of a statistically significant increase of the concentration of MMP1 and MMP3, which are responsible for collagen catabolism, and additionally GAG one, by MMP3 within these structure. The statistically significant decrease of collagen content observed within the cartilage in the case of allograft transplantation was associated with ECM homeostasis disorder of the cartilage as a response to critical increase of load, due to a decrease of meniscus allograft elasticity [6, 9, 17, 23]. Cartilage elasticity is the biomechanical property associated with proper distribution of load and shock absorption as well as load relief and protection of the subcartilaginous bone tissue, thanks to the GAGs, and the changes that we observed in the GAG content indicate the improper function of the cartilage after meniscus grafting [19, 24–31]. Our study confirmed the results by Temple-Wang et al. who disclosed that the increase of water content was concomitant with changes in the mechanical properties of the joint cartilage [32].

**Table 2:** The percentage decrease of water, glycosaminoglycans (GAGs), total collagen content, and decrease degree of elasticity in meniscus transplant [6] and cartilage in relation to control group

	Water (%)	Total collagen (%)	Glycosaminoglycans (%)	Degree of elasticity (%)
Cartilage autograft	↑ 6.8	↓ 0.7	↓ 13.4	↓ 14.8
Cartilage allograft	↑ 15.8	↓ 14.3	↓ 30.4	↓ 32.6
Meniscus autograft	↑ 4.9	↓ 0.4	↓ 14.9	↓ 19.5
Meniscus allograft	↑ 14.4	↓ 13.9	↓ 39.9	↓ 41.6

According to Armstrong and Mow, the studies evaluating the degenerative process of cartilage after meniscus transplantation, on the basis of macroscopic and histological assessment, are not reliable in evaluating its function and biological properties [20]. These observations are supported by Altman et al., whose study of degenerative disease in dogs revealed that changes in mechanical and biochemical properties occur earlier than histological alterations, which are regarded by these authors as secondary [18]. Although the performed study showed a statistically significant decrease of the degree of tibial cartilage elasticity, these were more pronounced in allograft transplants. These changes were found in the area of the cartilage covered by the meniscal transplant—the area in which Mikić et al. report degenerative changes to be the smallest [33].

The findings of the present study confirmed the results of Elliott et al. who revealed a decrease of the tensile modulus of the cartilage after meniscal transplants in dogs [34]. Bae et al. noted a reduced rigidity and increased softness of the femoral articular cartilage in the process of degeneration [35]. The Temple-Wang et al. report lowered Young’s modulus values of the cartilage during degenerative diseases [32]. The decrease of the elasticity degree of the cartilage after meniscus grafting carried out in the present research suggests further development of degeneration process in the cartilage. The presence of this process in the cartilage after meniscus transplants has been reported in numerous previous studies [9, 24, 27, 29, 30, 33, 36]. A long-term follow-up of human meniscus transplantation also indicates the development of osteoarthritis [2, 4, 36–38].

It is speculated, however, that on the basis of quantitative data obtained by the previous study and the current one, a decrease of meniscus elasticity exceeding 15 % can influence the homeostasis of cartilage ECM, manifested by an increase of apoptosis and MMP1 and MMP3 concentration, a statistically significant decrease in collagen and GAG content, and finally considerable reduction of its elasticity. Both this and previous studies indicate a close correlation between a decrease of GAG content and a decrease of elasticity within both the meniscus and the underlying cartilage. Furthermore, the average ratio of the decrease of cartilage elasticity to meniscus one was 0.77 regardless of the type of meniscus graft (Table 2) [6].

These results are vital for highlighting the relationship between the changes in the biochemical and biomechanical properties of meniscus and the texture of the cartilage they cover. In spite of a successful return to strenuous sporting activities after allograft meniscus transplantation, the data concerning the lower biological value of the allograft and its impact on the biochemical and mechanical properties of cartilage given in the present study indicate the necessity to critically revise the approach to treatment in this respect [1, 37, 38].

Moreover, collagen degradation after allograft indicates that this stage of the degenerative process of the cartilage might be irreversible and that sporting activities may accelerate its progression. In the present study, a decreased value of glycosaminoglycans/hydroxyproline ratio of the tibial cartilage was observed after both kinds of transplant (*p* < 0.05), the highest after allografts, what the degenerative process suggests [39].

Our present study shows that biomechanical and biochemical changes of the articular cartilage after meniscal autograft transplantation are less than after allograft. It suggests that the knowledge of chondroprotective effect of meniscal autograft could be useful for meniscus tissue engineering.

There are certain limitations of the present study due to the fact that its outcomes refer to the rabbit model. However, as the biochemical structure of the hyaline cartilage of the rabbit is very similar to that of human cartilage, it can be assumed that the changes occurring in the present experiment following meniscal transplantation can be related to humans. The mechanical investigations are also simplified and have been performed on the rabbit knee, which is of a small size. Moreover, the study of the cartilage was limited to the area covered by the meniscal grafts. Furthermore, in the case of the rabbit model, it is very difficult to perform a proper rehabilitation (human like) which may positively influence the final result compared to immediate full loading as in our study.

The results of both our previous and current study indicate that the autograft has considerably better molecular, biochemical, and biomechanical characteristics than the allograft and indicate how much must be done for meniscus transplant to bestow the chondroprotective effect or to form a scaffold similar to a viable autograft, which is still 15 % from the normal value of elasticity. The current stage of research is concentrated on searching the best stem cell material for repopulation of meniscus scaffolds, and the second stage is connected with the answer related to the quality of ECM composition they can produce. Finally, our results might provide some additional guidelines for the improvement of biochemical and biomechanical properties, and ultimately the chondroprotective abilities, of allografts and scaffolds used for meniscus tissue engineering [7, 40–46].

## Conclusions

1. The biochemical and mechanical properties of the type of viable meniscal graft have an influence on the biochemical composition of the ECM and the mechanical properties of the underlying tibial cartilage.
2. A decrease of 1 % of GAG content within the cartilage is connected with average 1.1 % decrease of its elasticity, regardless of the type of meniscus graft.
3. In contrast to autografts, the transplantation of viable meniscus allograft is connected with the development of irreversible ECM disorder of the cartilage.
4. The average ratio of the decrease of cartilage elasticity to that of the meniscus was 0.77 regardless of the type of meniscus graft.
5. Knowledge of the biochemical composition of the ECM meniscal grafts may serve as a predictor of their chondroprotective properties.
